# Adipose-Derived Mesenchymal Stromal Cells: A Tool for Bone and Cartilage Repair

**DOI:** 10.3390/biomedicines11071781

**Published:** 2023-06-21

**Authors:** Ivana Roberta Romano, Floriana D’Angeli, Nunzio Vicario, Cristina Russo, Carlo Genovese, Debora Lo Furno, Giuliana Mannino, Serena Tamburino, Rosalba Parenti, Rosario Giuffrida

**Affiliations:** 1 Department of Biomedical and Biotechnological Sciences, University of Catania, 95123 Catania, Italy; ivanarobertaromano@yahoo.it (I.R.R.); nunzio.vicario@unict.it (N.V.); cristina.russo@unict.it (C.R.); parenti@unict.it (R.P.); giuffros@unict.it (R.G.); 2 Department of Human Sciences and Quality of Life Promotion, San Raffaele Roma Open University, 00166 Rome, Italy; floriana.dangeli@uniroma5.it; 3 Faculty of Medicine and Surgery, “Kore” University of Enna, 94100 Enna, Italy; carlo.genovese@unikore.it; 4 Department of Chemical, Biological, Pharmaceutical and Environmental Sciences, University of Messina, 98122 Messina, Italy; 5 Chi.Pla Chirurgia Plastica, Via Suor Maria Mazzarello, 54, 95128 Catania, Italy; serenatamburino@hotmail.com

**Keywords:** adipose-derived mesenchymal stromal cells, stem cells, bone, cartilage, osteochondral defects, scaffolds, osteogenic differentiation, chondrogenic differentiation, regenerative medicine, tissue engineering

## Abstract

The osteogenic and chondrogenic differentiation ability of adipose-derived mesenchymal stromal cells (ASCs) and their potential therapeutic applications in bone and cartilage defects are reported in this review. This becomes particularly important when these disorders can only be poorly treated by conventional therapeutic approaches, and tissue engineering may represent a valuable alternative. Being of mesodermal origin, ASCs can be easily induced to differentiate into chondrocyte-like and osteocyte-like elements and used to repair damaged tissues. Moreover, they can be easily harvested and used for autologous implantation. A plethora of ASC-based strategies are being developed worldwide: they include the transplantation of freshly harvested cells, in vitro expanded cells or predifferentiated cells. Moreover, improving their positive effects, ASCs can be implanted in combination with several types of scaffolds that ensure the correct cell positioning; support cell viability, proliferation and migration; and may contribute to their osteogenic or chondrogenic differentiation. Examples of these strategies are described here, showing the enormous therapeutic potential of ASCs in this field. For safety and regulatory issues, most investigations are still at the experimental stage and carried out in vitro and in animal models. Clinical applications have, however, been reported with promising results and no serious adverse effects.

## 1. Introduction

In recent decades, regenerative medicine and tissue engineering have emerged with the purpose of rebuilding or repairing tissues and organs damaged by diseases, traumas or aging. In this context, a growing body of studies has been focused on stem-cell-based treatments for numerous human pathologies, including bone and cartilage disorders [1,2]. Stem cells are unspecialized cells capable of self-renewal and differentiation into various types of functional cells. There are different types of stem cells, such as embryonic stem cells, fetal stem cells, induced pluripotent stem cells and adult stem cells. The first three types are extensively studied for their marked proliferative potential and for their ability to differentiate into multiple cell lines; however, their use for clinical treatments is limited by considerable safety, ethical and regulatory issues [3]. For these reasons, much attention has been focused on adult stem or stromal cells that, although featuring lower differentiation capabilities, are more suitable for treatments in humans [4]. Adult stem cells are located in specialized niches of most tissues, where they continuously produce tissue-specific differentiated elements as well as other daughter stem cells that ensure a constant pool [5]. Hematopoietic stem cells in the bone marrow were the first population to be identified [6]. A few years later, a second population with different characteristics was discovered [7]. Defined as “bone marrow stromal cells” (BMSCs), they are a heterogeneous population of cells able to differentiate into mesodermal elements [8] and are thus considered mesenchymal stem cells (MSCs). Among these cells, marrow adipogenic lineage precursors seem to play important regulatory roles in bone remodeling, hematopoiesis regulation and marrow vasculature maintenance [9]. Under appropriate conditions, MSCs can differentiate not only into cells of the mesodermal lineage but also into elements of ectodermal or endodermal origin [10,11]. Other than bone marrow, MSCs can be isolated from numerous other tissues, such as the umbilical cord, dental pulp, skin, salivary glands [12,13] and from adipose tissue [14,15,16]. Comparative studies were carried out investigating the immunophenotype, proliferative potential, multilineage differentiation and immunomodulatory capacity of MSCs from different tissues (bone marrow, adipose tissue, the placenta and umbilical cord blood). On the basis of the gene expression profiles of stemness-related genes and lineage differentiation stage-related genes, it was found that no significant differences were observed in terms of the growth rate, colony-forming efficiency and immunophenotype. It was also found that BMSCs and ASCs shared both in vitro trilineage differentiation potential and gene expression profiles and represent the optimal stem cell source for tissue engineering and regenerative medicine [17]. In particular, adipose-derived MSCs (ASCs) will be considered in this review, as they have been widely studied for their numerous advantages. Unlike BMSCs, which require general anesthesia because of the painful harvesting procedure, ASCs can be obtained in large quantities with less invasive methods, and moreover, they feature even more proliferative activity and are more easily available for autologous administration [18,19].

## 2. Adipose-Derived Mesenchymal Stromal Cells (ASCs)

ASCs are commonly isolated from the stromal vascular fraction (SVF) of adipose tissue, which is diffusely located in the human body at either the subcutaneous or visceral level. Depending on the anatomical region from which the adipose sample is taken, some differences have been reported. For example, a greater number of cells can be isolated from the adipose tissue of the arm, whereas cells with excellent plasticity can be obtained from the groin area [20]. Many studies have been carried out on adipose tissue contained in the lipoaspirate that is available after liposuction and would be otherwise discarded. Although the harvesting procedure is generally accurate, contaminations may occur. Specifically, microorganisms (bacteria, viruses and fungi) can infect samples during the various preparation steps so that infections in the host tissues and immune reactions might be generated after implantation [21]. For this reason, microbiological control is fundamental at each stage to ensure the absence of contamination and the safety of the final product. Once isolated, ASCs can be expanded and identified by their plastic adherence, colony forming capacity and rapid proliferation. They show a fibroblast-like morphology and are positive for typical MSC markers (CD44, CD73, CD90 and CD105) but not for typical hematopoietic markers (CD14, CD34 and CD45). According to their MSC nature, they are able to differentiate toward osteogenic, adipogenic, myogenic and chondrogenic lineages [22,23,24,25]. However, with specific treatment, ASCs can also differentiate not only into other cells of mesodermal derivation [26,27,28] but also into cells originating from endodermal or ectodermal embryonic layers, such as neural cells [29]. This can usually be obtained by supplementing the growth medium with bioactive molecules, although satisfactory results can be achieved by using conditioned media from other cell cultures [30,31,32,33,34,35]. It is important to underline that ASCs have a low immunological reactivity, thanks to the absence or low expression of immunogenic surface antigens (CD40, CD40L, CD80 and CD86) and major histocompatibility complex II. This low immunological reactivity also makes them suitable for allogeneic use [20]. In addition, ASCs are able to modulate T and B cell activity and exert anti-inflammatory effects [36]. Finally, ASC beneficial effects can also be due to their paracrine production of numerous cytokines and growth factors, such as vascular endothelial growth factor (VEGF), fibroblast growth factor (FGF) and insulin-like growth factor 1 (IGF-1) [37,38]. As a result, the use of an ASC-conditioned medium is also investigated for potential cell-free therapeutic applications [39].

### 2.1. ASC Osteogenic Differentiation

The osteogenic induction of ASCs is usually obtained in about 21 days by replacing the basal growth medium with osteogenic media containing dexamethasone, β-glycerophosphate and ascorbic acid [40]. To verify the osteogenic phenotype, specific histological staining is commonly used (Von Kossa or Alizarin Red), which reveals calcium deposits and the mineralized matrix [1]. During differentiation, ASCs produce a mineralized extracellular matrix (ECM) with an increased expression of bone markers, such as runt-related transcription factor 2 (RUNX2; a marker of osteoblast differentiation), osteonectin and osteocalcin, as well as an increased synthesis of alkaline phosphatase (ALP) and collagen type I [41,42]. A better degree of osteogenic differentiation has recently been obtained using ASCs transfected with osteo-specific genes, such as the insertion of encoding bone morphogenetic protein (BMP)-2 and Runx2 genes [43]. Controversial results were reported by comparing ASC and BMSC osteogenic potential. According to Brennan et al. [44], BMSCs show better osteogenesis, whereas ASCs feature enhanced angiogenesis. In contrast, in another comparative study, it was found that ASCs showed a higher proliferation rate and an increased ability to differentiate into osteocytes and chondrocytes, as evaluated using Alizarin Red and Alcian Blue staining, respectively [18]. In particular, these properties were more pronounced for cells expressing CD271, a neurotrophin receptor that is a member of the tumor necrosis factor (TNF) receptor superfamily. However, the differences might be simply due to different experimental procedures.

### 2.2. ASC Chondrogenic Differentiation

Various protocols have been adopted to obtain ASC chondrogenic differentiation, normally requiring 21–28 days [45]. Widely used differentiation media usually contain high glucose levels, fetal calf or bovine serum, penicillin and streptomycin, ascorbate-2-phosphate, dexamethasone, transforming growth factor beta (TGF-β1 or TGF-β3), insulin, transferrin, selenium, sodium pyruvate and L-glutamine. Additional supplements and growth factors can be included, such as BMP-2, 4 or 6, sex-determining region Y box 9 (SOX 9) and basic FGF [46,47,48]. The chondrogenic phenotype can be evaluated using various tests, including histological staining using Alcian blue, toluidine blue or safranin O, detecting the presence of proteoglycan in the cartilage-like matrix. In addition, real-time PCR, Western blot analysis, ELISA, RNA microarray analysis and immunohistochemistry reveal the expression of chondrocyte-specific genes or proteins, such as different collagen types, keratin sulfate, chondroitin sulfate, aggrecan, decorin and biglycan [41,49]. In monolayer cultures, ASCs hardly maintain a chondrogenic phenotype, showing a decrease in collagen type II expression, while collagen type X production increases. A three-dimensional (3D) culture of chondrocyte-like ASCs overcomes this issue. In a study by Musumeci et al. [50], ASCs treated with a specific chondrogenic medium for 28 days showed a high expression of collagen type I and II, as well as lubricin, a key molecule in cartilage wear prevention. This glycoprotein, which assures joint lubrication and synovial homeostasis, may represent an additional differentiation biomarker.

## 3. ASC-Based Repair Strategies

**Figure 1:**
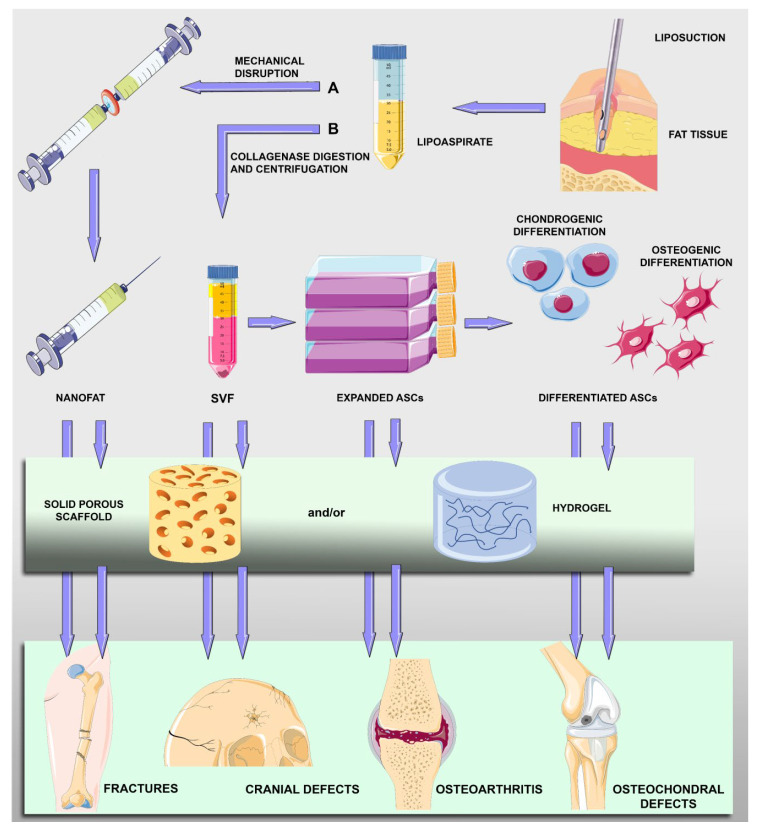
Schematic drawing showing main steps of harvesting and treatments of adipose-derived mesenchymal stromal cells (ASCs) for bone and cartilage repair. After liposuction procedures, fat tissue in the lipoaspirate can be mechanically disrupted (**A**) to obtain micro fat particles (nanofat) containing ASCs and other cell types (lymphocytes, pericytes, vascular endothelial cells and vascular smooth muscle cells). Lipoaspirate can be digested and centrifuged (**B**) to obtain a pellet (stromal vascular fraction, SVF) containing ASCs that can be expanded and undergo osteogenic or chondrogenic differentiation. Nanofat particles, SVF cells, expanded ASCs or predifferentiated ASCs can be directly implanted or included in scaffold-assisted treatments.

Different approaches have been explored for the treatment of cartilage and bone tissue disorders: the transplantation of uncultured ASCs; the transplantation of ASCs expanded in vitro; the transplantation of ASCs after their osteogenic or chondrogenic differentiation; and variously treated ASCs in combination with scaffolds (Figure 1).

For cell-based applications, freshly isolated ASCs present in adipose SVF are preferred in some instances, not only to shorten the interval from harvesting to the transplant procedure but also to avoid restrictions and risks related to cell culturing, such as the possibility of contamination, tumorigenesis or unexpected cell differentiation. A collagenase enzyme is normally added to the adipose tissue, producing a mixture that, after filtration and centrifugation, can be separated into a superficial adipocyte fraction and precipitated cellular components. To overcome restrictions associated with the enzymatic procedure, alternative methods to obtain an SVF-like compound have been developed. Although with a lower cell yield, SVF cells featuring virtually the same regenerative potential can be obtained using mechanical disruption of the adipose tissue giving rise to micro fat particles that are also called nanofat [51,52]. Nanofat grafting is widely used in plastic surgery [53] and for bone and cartilage repair, often combined with platelet-rich plasma (PRP) [16]. After centrifugation, ASCs contained in the SVF can be successively expanded for further uses. Based on cell–cell and cell–matrix interactions, 3D cultures of ASCs would offer a favorable context for their multilineage vascular and osteogenic differentiation, providing in the resulting spheroids an in vitro model of the interactive elements [54]. This strategy is particularly advantageous to improve the thickness of a scaffold-free implant. Figure 1 illustrates the main steps of ASC treatment for bone and cartilage repair.

### Scaffold-Assisted Strategies

Scaffold-based approaches have been widely investigated as cell implantation alone often raises substantial issues related to postimplantation cell fate, cellular loss and dispersion. A variety of scaffolds have been designed, improving not only the correct positioning of stem cells but also their attachment, viability, proliferation, migration and differentiation [55,56,57]. Scaffolds can be subdivided into different categories (i.e., metallic or nonmetallic, natural or synthetic), and many of them are available for applications to skeletal and other tissues. Due to their properties, metallic implants using titanium and titanium dioxide are particularly suitable for bone defects. On the other hand, many more nonmetallic scaffolds have been designed, each of them characterized by different advantages and disadvantages. Examples of these scaffolds include the acellular matrix, coralline scaffolds, natural or synthetic polymers and hybrid scaffolds [20,58,59]. Ideal scaffolds should be degradable and biocompatible and mimic as closely as possible the physiological microenvironment of the target tissue. In addition, they can be loaded not only with stem cells but also with bioactive molecules, such as growth factors and anti-inflammatory or antibacterial agents [60]. Optimal pore size is a crucial characteristic of a scaffold and largely depends on the type of tissue for which the scaffold is planned [61]. For example, smaller pore sizes are suitable for the formation of fibrous tissues, while larger pore sizes are more appropriate for the formation of bone tissue. Too wide pores hamper cell attachment and the stiffness of the scaffold, whereas too small pores reduce cell viability and migration, nutrient diffusion and waste removal. After the initial adhesion and tissue formation at the external surfaces, stem cells should be able to penetrate inside the scaffold giving rise to an inward gradient of tissue formation [62]. The newly formed tissue would hopefully restore the proper shape and function.

Natural ECMs can be advantageously prepared in the form of hydrogels [58], which are considered a promising cell-supporting alternative material [63]. Hydrogels are characterized by a 3D polymeric network that, retaining a large amount of water, more closely resembles soft tissues, thus providing a wider range of scaffold applications. Hydrogels possess good biocompatibility, tunable swelling and mechanical properties and a biodegradation rate that is more predictable and adjustable than other scaffolds. The most used natural biomaterials include collagen, gelatin, hyaluronic acid, chitosan and alginate, noncollagenous proteins and proteoglycans [64,65].

For in vivo experiments, rodents are mostly investigated, but larger animals (goats, dogs or minipigs) are also often chosen because a large animal requires implantation procedures that are more similar to those for human treatments, thus improving the translational potential.

## 4. Bone Repair

ASC-based strategies have been tested to address various bone disorders, such as fractures, cancer-related bone resections, cranial and craniofacial defects, osteoporosis or other disorders. In the case of bone fractures, although successful bone healing and recovery is usually achieved, sometimes (5–10% of cases) delayed or nonunion healing occurs, especially in cases of comorbidities, such as diabetes [66]. Autologous bone grafting or bone tissue from compatible donors is generally considered the best choice. However, the small volume of bone that can be harvested (usually from the iliac crest) for subsequent transplantation considerably restricts this approach to small-size defects. For large defects, autologous grafting shows some significant limitations: the harvesting procedure might be technically difficult, it has a risk of donor site morbidity, and the transplanted bone may fail to integrate into the host site. For these reasons, other strategies may be usefully adopted. For example, synthetic bone grafts can be loaded with bioactive molecules to stimulate bone formation using native precursor cells or supplemented with ASCs. As mentioned above, beneficial ASC effects may rely not only on their ability to differentiate into osteogenic lineage elements but also on their release of soluble factors and extracellular vesicles that can improve proliferation and the activity of resident osteoblasts, as has been demonstrated in vitro [67]. To date, many experiments have been carried out in animal and in vitro models, and clinical trials confirm their regenerative potential.

ASCs engineered to overexpress basic FGF were tested on the fracture healing of mice femurs after intramuscular cell injections within or around the fracture site [68]. These engineered ASCs were able to migrate and engraft in the newly formed callus, also promoting collagen remodeling into mineralized callus and bone. In addition, an increased VEGF expression was observed in the periosteal region of the callus. It was concluded that these engineered ASCs accelerated fracture repair by stimulating angiogenesis and ASC osteoblastic differentiation. This is not surprising as a vascular disruption is often a direct consequence of fractures, and it is recommended that osteogenic processes are simultaneously accompanied by increased blood vessel support. In fact, ASCs contain vasculogenic subpopulations that promote neovascularization [39], thus providing an added benefit. In this regard, a promising method could be the administration of ASCs combined with endothelial cells. In vitro experiments demonstrated that cocultures of human umbilical vein endothelial cells and osteogenic-differentiated ASCs significantly improved cell survival and proliferation, osteogenesis and angiogenesis. In these preparations, matrix mineralization and the activity of ALP was increased, as well as the gene expression of proangiogenic markers. The enhanced angiogenic potential was confirmed by the number and length of endothelial tubular formations in 3D Matrigel cultures [69]. In a study by Wang et al. [70], some ASCs were preliminarily cultured to form an osteogenic cell sheet and, combined with some others, induced to differentiate into endothelial progenitor cells. This assembly was then subcutaneously implanted into nude mice and compared with implants of osteogenic cell sheets alone. After 8 weeks of survival, a new denser ectopic bone tissue was formed when the osteogenic sheet was combined with endothelial progenitor cells, also showing more numerous vascular structures. When these complexes were implanted in rabbit calvarial defects, better bone reconstruction was observed.

Osteoporosis commonly results from estrogen deficiency in postmenopausal age because of the imbalance between bone resorption and bone formation. In a mouse model of osteoporotic animals undergoing ovariectomy, the proliferation rate and osteogenic differentiation of ASCs was compared with ASCs derived from control mice [40]. After osteogenic induction, ASCs from osteoporotic animals showed a lower proliferation rate and a lower capacity to form mineralized nodules. It was concluded that, in cases of autologous transplantation, these ASCs would need improved osteogenic predifferentiation. The influence of donor age was tested in a comparative study between ASCs isolated from young and aged mice. In vitro experiments showed that ASCs from young mice exhibited the typical fibroblast-like spindle shape, whereas those from aged animals were larger, showing a flat irregular shape. Moreover, aged ASCs revealed a significant decline in both osteogenic differentiation potential and proliferation rate. When transplanted into the bone marrow of osteoporotic mice, it was found that after 4 months from injection, a significantly higher bone regeneration was induced by young ASCs, along with an increased bone mineral density and enhanced mRNA levels of osteogenic markers (RUNX2 and osteopontin) [71]. The effect of local injections of BMSCs and/or ASCs was evaluated on the regeneration of rat calvarial defects [72]. It was shown that these treatments resulted in increased bone formation compared to the controls. Both cells had the ability to induce bone tissue formation at comparable amounts, and their association enhances bone repair properties [73].

### Scaffold-Assisted ASC Implantations

The combination of ASCs with collagen/Mg-doped hydroxyapatite scaffolds has been investigated [42]. Through Alizarin Red staining and gene expression profile analysis, it was found that even in the absence of specific inducing factors, ASCs were able to differentiate into mature osteoblasts, as suggested by the expression of specific osteoblast and ECM markers. However, the osteogenic process was markedly accelerated by the presence of osteoinductive factors. After subcutaneous implantation in the back of mice [74], it was confirmed that these scaffolds could promote ectopic bone formation; however, better results were obtained by the addition of ASCs, especially after 4 and 8 weeks of survival. In further experiments, an excellent antibacterial property was achieved by adding silver nanoparticles to Mg–hydroxyapatite scaffolds [75]. The effects of TNF-α were tested on ASC-seeded polycaprolactone (PCL)–fibrin composite scaffolds [76]. In fact, it has been shown that this proinflammatory cytokine may improve bone formation and angiogenesis in a dose- and time-dependent manner. After in vitro pretreatment with TNF and/or platelet-derived growth factor (another key factor released following bone injury), ASC-seeded scaffolds were subcutaneously implanted in athymic nude rats for 2 weeks. The results showed an increased staining for collagen I and osteocalcin when compared with the preimplantation stage, especially after TNF treatment. In addition, costimulation with platelet-derived growth factor synergistically increased the vascular network formation. The proangiogenic effects of PRP were confirmed analyzing results obtained after its supplementation to ASC-containing alginate microspheres with osteogenic and angiogenic potential [77]. Subcutaneous injections in athymic mice showed that this combination was able to improve the blood vessel network and significantly increased mineralization. In another study, the subcutaneous implantation of ASCs obtained from bidimensional cultures and ASCs seeded in 3D PCL scaffolds were tested in immunodeficient mice [78]. The results showed that an upregulation of osteogenic markers (RUNX2, collagen I, ALP, osteonectin and osteocalcin) was improved in the 3D scaffolds, and no significant differences could be observed when tricalcium phosphate (TCP) was added.

Other scaffold types have, however, been developed. For example, improved ASC osteogenic differentiation was reported for functional polymer scaffolds with polydopamine-assisted BMP-2 immobilization [79], and enhanced bone formation was also described for ASCs encapsulated in methacrylated gelatin hydrogels supplemented with BMP-2 [80]. Three-dimensional constructs for implantation in bone defects were created in vitro by embedding predifferentiated ASCs in a collagen matrix placed in a microfluidic chip [81]. The authors claim that this “bone-on-a-chip” device represents an intermediate step between traditional in vitro and in vivo experiments, therefore reducing experiments in animals. In addition, mimicking the bone microenvironment, it can improve ASC differentiation toward bone cells and can be personalized for specific patients.

In both humans and animal models, coral exoskeleton has been used as a scaffold to treat bone defects since the early 1970s. Coral scaffolds are biocompatible, osteoconductive and bioresorbable and feature a 3D pore architecture similar to that of human bone [82]. When implanted in vivo, coral scaffolds containing cells and/or growth factors induce a significant increase in newly formed bone tissue. In a study by Wang et al. [83], ASCs isolated from the inguinal adipose tissue of rabbits were cultured for two weeks under osteogenic induction and seeded on coral scaffolds where they were able to proliferate. These osteoblastic-sheet–coral complexes were implanted into subcutaneous pockets in nude mice and examined at eight weeks after implantation. Gross examination, microcomputed tomography and histological analysis demonstrated that a denser tissue was formed in these animals when compared with the control group.

In cases of fractures and in other bone defects, titanium-based implants are widely used. To optimize their efficacy and to achieve a better integration of the metallic implant with the surrounding tissue, metallic implants can be combined with various materials, such as drugs, silicon dioxide, hydroxyapatite or stem cells. An improved osteogenic activity around implants has been recently reported by using titanium dioxide porous three-dimensional scaffolds with high biocompatibility and osteoconductivity for the treatment of large bone defects [84]. ASC-supplemented 3D-printed titanium scaffolds were tested in a rat model with a full-thickness mandibular defect [85]. In the first group, cultured ASCs were added to the scaffold after its implantation; in the second group ASCs cultured in cell matrix hydrogel were impregnated into the metallic scaffolds before implantation; and the third group, where only the titanium scaffold was implanted, served as the control. At 12 weeks after surgery, the best osteogenic differentiation and new bone formation were detected in animals implanted with scaffolds and ASCs cultured in cell matrix hydrogel; only minor new bone formation was observed for the implantation of the other ASC-treated scaffold, and almost no bone formation was found in the control group. In dogs with mandibular defects, ASC aggregates were injected into artificial 3D-printed PCL/TCP scaffolds coated with demineralized and decellularized bone ECM [86]. Compared with animals implanted with acellular scaffolds, the results obtained after 4 and 8 weeks of survival showed a more diffuse osteoblast presence along with rich ossification in the scaffold pores. In addition, enhanced collagen I, osteocalcin and Runx2 gene expression were assessed using real-time PCR, and a greater expression of corresponding proteins was revealed through Western blotting. Injections of ASCs in a solution of human PRP and tail collagen in a mandibular osteoradionecrosis model of athymic rats increased bone deposition and preservation [87]. Ulnar bone defects in minipigs were implanted with an acellular bone matrix supplemented by autologous ASCs genetically modified to release BMP-2 and VEGF [88]. Through X-ray, radionuclide bone imaging and SPECT examinations, the therapeutic effects were evaluated 2, 4, 8 and 12 weeks after the intervention. It was found that significantly accelerated bone formation could be observed in treated animals when compared with the controls.

For substantial cranial defects, ASCs in combination with polylactic acid (PLA) scaffolds were tested in a rabbit model [89]. For this purpose, six combinations of implants were comparatively studied: (1) PLA alone (control); (2) fibronectin-coated PLA; (3) PLA with ASCs; (4) fibronectin-coated PLA with ASCs; (5) PLA with osteogenically induced ASCs; and (6) fibronectin-coated PLA with osteogenically induced ASCs. After 6 weeks of survival, X-ray, histology and histomorphometric analyses showed that no bone formation could be found in the control group, whereas different degrees of new bone deposition were present in the other groups. The best results were observed in animals implanted with fibronectin-coated PLA scaffolds supplemented with osteogenically induced ASCs. Gelatin/VEGF-coated poly(ɛ-caprolactone) scaffolds containing ASCs transduced with lentiviruses expressing osterix were implanted into rat calvarial critical-sized defects, leading to more bone formation than other scaffold types [90]. In another mouse model of cranial defect, injectable and in situ cross-linkable gelatin microribbon-based macroporous hydrogels were tested for supporting ASC delivery and bone regeneration [91]. The results obtained showed good levels of bone formation, especially if BMP-2 was added to the scaffold. Indirect cocultures of ASCs and osteoblasts were arranged in vitro in a collagen-based 3D scaffold. Likely due to osteoblast paracrine activity, osteogenic differentiation of ASCs occurred. When implanted in rat calvarial defects, the results were compared with other groups of animals where monocultured ASCs or ASCs supplemented with BMP-2 were transplanted. Overall, a higher level of bone formation and coverage ratio was found in animals where ASCs were associated with osteoblasts or BMP-2, whereas poorer outcomes were obtained after ASC treatment alone [92]. Photobiomodulation was found to be effective in enhancing in vivo bone regeneration and the osteogenic differentiation of ASCs encapsulated in methacrylated gelatin hydrogels [93]. Implantations of these complexes were carried out in rats with biparietal bone defects, and polychromatic light was administered at 20 cm distance for 10 min in each session in 48 h intervals. Comparative analyses were carried out in three groups of animals: animals with blank defects (controls), with or without light administration; animals with acellular methacrylated gelatin implantation, with or without light administration; and animals with ASC-methacrylated gelatin implantation, with or without light administration. After 20 weeks, the calvaria were harvested for macroscopic, microtomographic and histologic evaluations. Compared with the control group, better results were obtained in the animals implanted with the methacrylated gelatin hydrogels, especially when ASCs were also encapsulated, showing the highest score for mineralized matrix formation. However, between animals of each group (with or without light administration), better results were consistently observed in those receiving light administration, indicating the regenerative effect of photobiomodulation.

ASC-derived exosomes coupled with ECM hydrogel were tested in a rat model of intervertebral disc degeneration, a chronic degenerative disease characterized by a reduction in collagen type II and increased ECM catabolism caused by an abnormal increase in metalloprotease activity. It was found that ASC-derived exosomes could slow down ECM catabolism by reducing the activity of metalloproteases, thus promoting ECM regeneration [94].

Although with considerable restrictions, some clinical trials have been carried out to assess the safety of implantation procedures and ASC ability to restore bone tissue. For example, a scaffold-free osteogenic 3D graft was implanted in patients for the treatment of bone nonunion [95]. To create the 3D implant, cultured autologous ASCs were added to demineralized bone matrix, well known for its osteoinductive properties. The graft was opportunely modeled to fit the bone defect and placed without any fixation material into the bone gap. The authors reported encouraging results without serious adverse events for up to 54 months. A similar 3D graft, in which ASCs were preincubated in an osteogenic medium and added to demineralized bone matrix, was tested in patients with long bone nonunion or tumor resection [96]. On the basis of the results obtained, the authors claim that this engineered tissue can safely promote osteogenesis, restoring bone functionality with no oncological side effects and minor donor site morbidity.

The main results of the ASC-based strategies are summarized in Table 1.

## 5. Cartilage Repair

In the skeletal system, cartilage is responsible for two main functions as articular cartilage at bone extremities and as a scaffold during endochondral ossification [97]. Articular cartilage is an avascular connective tissue in which chondrocytes are embedded in small cavities (lacunae) and surrounded by a dense ECM containing proteoglycans and collagen type II. The particular ECM composition and organization contributes to the mechanical properties of this tissue, which is essential for smooth/painless joint movements, and acts as a biomechanical shock absorber. Due to its avascular nature, injured articular cartilage possesses a very limited self-repair capacity. Cartilage damage may be caused by aging, physical trauma, infection or various diseases, such as osteochondritis or osteoarthritis (OA). OA not only affects the articular cartilage but also most tissues within and surrounding the joint (ligaments, joint capsule and synovial tissue). Synovial fibroblasts are mesenchymal-derived cells that produce synovium, a lubricating fluid that also supplies nutrients to articular chondrocytes. Pharmacologic therapies for OA mainly manage the symptoms and consist of analgesics and anti-inflammatory drugs, but none of these effectively reduce disease progression. Several therapeutic approaches have been aimed at hyaline cartilage regeneration, but to date, there is no efficacious procedure to restore its mechanical and functional properties. Traditional surgical methods include microfracture surgery [98] or arthroscopic drilling [99], with the purpose of stimulating resident cell proliferation and differentiation into chondrocytes. However, the newly formed tissue often consists of unsuitable fibrocartilage [100]. At late stages, total joint replacement and metal resurfacing is the only treatment that can provide satisfactory pain relief and function recovery. The transplantation of autologous chondrocytes may produce some beneficial effects, but this approach is restricted by a number of factors: the limited availability of chondrocytes, damage at the donor site after harvesting procedures, and reduced survival of the transplanted cells [49]. Although other cell types do not completely offer results comparable to those of original chondrocytes, because of their multipotent ability, ASCs are considered a valuable alternative.

Adipose SVF combined with PRP was injected into the knee of mice after cartilage damage [101], and compared to control animals without cell transplantation, this treatment significantly attenuated the cartilage defect, and the animals could use the injured hind limb in a shorter time. Indeed, histological observations confirmed that increased neocartilage formation could be detected. The authors also report that the expression of cancer-related genes, such as Oct-3/4 and Nanog, was much lower than in embryonic stem cells. Another study was carried out on a rabbit model of OA induced by the unilateral transection of the anterior cruciate ligament. In these animals, a single dose of medium containing suspended ASCs was injected in the knee at 12 weeks following surgery, whereas only medium or nothing was injected into two other control groups [102]. After 16 and 20 weeks of survival, radiological and histological analyses revealed significant improvements in the quality of cartilage in ASC-injected animals compared with the controls. Experimental genetic modifications were induced in mouse ASCs, which were transfected with chemically modified mRNA to secrete trophic factor IGF-1 [103]. Preliminary in vitro experiments showed that these engineered ASCs significantly increased their paracrine actions and the expression of chondrocyte anabolic markers. When injected in the knee of a mouse model of OA, after surgical destabilization of the medial meniscus, it was found that (a) the survival rate of IGF-1-ASCs increased, (b) their transplantation markedly prevented cartilage degeneration, and (c) they effectively restored ECM deposition, as assessed by a higher expression of aggrecan and collagen type II. Native ASCs and chondrocyte-like differentiated ASCs were tested in a rat model of OA obtained using the transaction of the anterior cruciate ligament and resection of the medial meniscus [49]. The results were gathered 4 weeks after cell administration in the joint under the patellar tendon. Compared with the normal control group, characterized by smooth cartilage with even margins where chondrocytes were uniformly distributed, OA knees showed severe proteoglycan loss, the formation of osteophytes and fibrillation. In animals with injections of ASCs or predifferentiated cells, a marked reduction in fibrosis was found, along with the formation of hyaline-like neocartilage. Overall, better results were obtained after the transplantation of chondrocyte-predifferentiated ASCs.

### Scaffold-Assisted ASC Implantations

Freshly isolated adipose SVF and cultured ASCs were comparatively tested in an in vivo study on Dutch milk goats, where cartilage defects were created in medial condyles and trochlear grooves of the knee [104]. In some defects, SVF or ASCs were implanted using collagen I/III scaffolds, whereas analogous acellular constructs were implanted in other similar defects for comparative evaluations. After 4 months, the regeneration processes observed in sites where cell-free constructs were implanted were less developed than those detected for cell-containing constructs, which were characterized by a more extensive expression of collagen type II, hyaline-like cartilage and a better integration of glycosaminoglycan with the host tissue. Moreover, higher levels of regenerated subchondral bone were found, showing more intense collagen type I staining. Overall, better results were tendentially obtained after SVF treatment, inducing the authors to hypothesize two possible explanations: (a) other than ASCs, SVF includes other cell types that may exert synergistic effects at later stages; and (b) noncultured ASCs present in SVF feature a higher differentiation potential than cultured cells. For these reasons, also taking into account economic and regulatory issues, the use of SVF would be more suitable. In fact, adipose SVF was recently used to alleviate pain and improve the knee function of OA patients. Moreover, SVF may induce beneficial effects by modulating inflammation and improving paracrine activity that slow down degeneration and stimulate joint tissue regeneration [105]. Percutaneous injections of ASCs combined with hyaluronic acid, PRP and calcium chloride were performed in the knee of OA patients and into the femoral heads of osteonecrosis patients [106]. MRI data confirmed cartilage regeneration and bone formation for the two groups of patients. Single intra-articular injections of autologous ASCs were performed in France and Germany after regulatory agency approval for ASC expansion procedures [107]: different ASC doses were injected into three groups of patients with severe knee OA. Although in the absence of placebo-treated controls, the procedure was found to be safe, and no serious adverse events were reported at 6 months of follow-up. The authors report that the best results were observed in patients treated with low-dose ASCs, showing significant improvements in pain relief and joint function. Satisfactory improvements and pain relief were also reported for patients with knee osteoarthritis after intra-articular injections of autologous ASCs [108].

Cartilage-based scaffolds are often preferred as they contain original ECM components that may provide useful cues for cell proliferation, attachment and differentiation. In fact, ASCs seeded on cartilage-derived particles more easily differentiate into chondrocytes without the addition of growth factors. Rabbit ASCs were isolated, cultured and seeded in the cartilaginous matrix of the same animal to undergo in vitro chondrogenic differentiation for 2 weeks [100]. This engineered ECM was then transplanted into cartilage defects in the knee to evaluate results after 12 weeks of survival. It was found that cartilage defects in these animals were filled with chondrocyte-like tissue with a smooth surface. The histological and immunohistochemical analyses of the restored tissue showed positive Alcian blue staining and collagen type II expression, similar to normal cartilage. In addition, it was seen that these cells migrated to the inner material, attached, proliferated and differentiated into chondrocytes. To some extent, the repaired defect allowed a recovered joint function. In contrast, the cartilage defect was filled only with fibrous tissue in another group of animals where only the acellular matrix was implanted, and no repair tissue was found in a further animal group with no implantations. Similar results were also reported after experiments in a rabbit model where knee cartilage was damaged [109]. In brief, good levels of hyaline cartilage regeneration were observed after the implantation of ASC-loaded cartilage-derived scaffolds, whereas only fibrous tissue was generated following the implantation of analogous acellular scaffolds. In another study, biodegradable porous sponge cartilage scaffolds were used to test the regeneration of hyaline-like cartilage in rabbits where femoral trochlea cartilage was damaged [110]. The implantation effects of cartilaginous scaffolds supplemented with ASCs or their secretome were compared with other animal groups where only scaffolds or nothing was implanted. Observations after 12 weeks of survival revealed that the mean score for all implanted groups was better than the control group of nonimplanted animals. Macroscopic and microscopic evaluations showed that the addition of ASCs was better than secretome in enhancing cartilage regeneration.

ASCs isolated from the adipose tissue of the iliac fossa were cultured and mixed with calcium alginate gel to be implanted in full-thickness hyaline cartilage defects created at the patellofemoral joint of rabbits [111]. Only acellular gel or nothing was implanted in the control groups. The histological analysis and qualitative scoring at 4, 8 and 12 weeks showed that cartilage defects were completely repaired only in the group with ASC implantation, whereas only fibrous reconstruction tissue was mostly present when the acellular gel or nothing was implanted. Intra-articular injections of ASCs seeded in an amnion-membrane-based biomimetic injectable hydrogel were tested in a collagenase-induced OA rat model [112]. At one week from OA induction in the knee, different injections were performed in four groups of rats: hydrogel–ASCs, hydrogel alone, ASCs alone and phosphate-buffered saline as the control. Observations at 2 and 3 weeks from injections revealed that knee swelling in the hydrogel–ASC group was significantly lower (indicative of a decreased synovial inflammation) compared with the controls, hydrogel, and ASC groups. Cytokine profiling, Raman spectroscopy and histology also confirmed the synergistic anti-inflammatory and chondroprotective effects of this composite hydrogel.

The main results of the ASC-based strategies are summarized in Table 2.

## 6. Osteochondral Defect Repair

Articular cartilage and the underlying bone are continuously subjected to the biomechanical stress associated with movement and loading so that articular cartilage relies on the subchondral bone to maintain its homeostasis and integrity [113]. Through in vitro experiments, it was found that the absence of subchondral bone could lead to chondrocyte death in a few days, whereas they remained viable when cultured in its presence, likely due to the bone release of survival factors [114]. In this respect, the calcified cartilage layer may represent a barrier between the subchondral bone and the articular cartilage [115], although it has been reported that communication between the two compartments may occur either through blood vessels traveling from bone into the cartilage or areas of uncalcified cartilage. Consequently, an altered crosstalk between the articular cartilage and the underlying bone would affect the entire region producing wider disorders also known as osteochondral (OC) defects [116]. These usually develop into OA and are characterized by cartilage degradation and subchondral bone alterations. For the treatment of OC defects, the use of ASCs has been widely explored, either by developing scaffold-free or scaffold-supported cell implantations. However, substantial difficulties remain in obtaining the simultaneous regeneration of the superficial hyaline cartilage and the underlining subchondral bone, a characteristic that is not normally assured by a general scaffold.

The regeneration of articular cartilage and subchondral bone was tested in a pig model using scaffold-free 3D constructs of ASCs [117,118]. To this purpose, two cylindrical osteochondral defects were created in the patellofemoral groove of one side. A columnar structure of autologous ASC spheroids was assembled by placing approximately 770 spheroids into a cylindrical mold (5 mm diameter) and incubated in a complete culture medium until their fusion. A cylindric construct was implanted into one defect, whereas the other was used as a control. After 6 months from implantation, active endochondral ossification was revealed underneath the fibrocartilage in the implanted defects, whereas a modest fibrocartilaginous coverage was observed in the controls. After 12 months, the fibrocartilage was converting into hyaline cartilage with the same thickness as the surrounding native cartilage, along with the regeneration of the subchondral bone. An analogous strategy was successfully adopted implanting allogeneic ASC columnar constructs into the OC defects of rabbits [119]. The authors report that after 12 weeks the implanted cells survived, adhered to the defect and regenerated articular cartilage and subchondral bone. A more sophisticated autologous ASC construct was developed by Yamasaki et al. [120] to be implanted in columnar OC defects at the center of the groove in both the hind limbs of minipigs. Briefly, ASC-derived spheroids were arranged to form two cylinders: one solid cylinder inserted into an empty cylinder. The internal solid cylinder was created by piling ASC spheroids into a “mold” made of Teflon where they eventually fused. The external empty cylinder was obtained by using the “Kenzan” bioprinting method, where spheroids were placed in a circular needle-array-arranged device to form the cylinder wall. The “Kenzan”, which plays the role of a temporary support, was later removed after spheroid fusion before implantation. This concentric columnar construct was then grafted into the OC defect in the right knee, whereas no graft was implanted into the contralateral one. On the basis of results obtained, the authors conclude that implantation of this scaffold-free artificial construct of ASCs can support tissue regeneration in OC defects.

### Scaffold-Assisted ASC Implantations

An artificial 3D bilayered osteochondral construct supplemented with ASCs was designed by Song et al. [121] to treat large OC defects. In this composite scaffold, porcine cancellous bones and chitosan/gelatin hydrogel were arranged at the two opposite sides to induce the regeneration of bone tissue and cartilage, respectively. To this purpose, chondrocyte-like ASCs were seeded in the hydrogel, and osteoblast-like ASCs were seeded in cancellous bones. The authors report that, likely due to intercellular interactions, this bilayered scaffold significantly enhanced ASC proliferation compared to cells seeded on either single scaffold. A simultaneous regeneration of hyaline-like cartilage and subchondral bone was attempted in a rabbit model of OC defects by using poly(l-glutamic acid)-based bilayer scaffolds supplemented with autologous ASCs [122]. In these scaffolds, two different regions were processed differently at the two opposite sites to support the hyaline cartilage and underlying bone regeneration. Before implantation, by creating opposing gradients of bioactive signals, ASC osteogenic differentiation was induced in the lower part of the scaffold by the presence of abundant BMP-2, whereas superficial chondrogenic differentiation was induced by TGF-β1 and IGF-1 for 7 days in the upper part of the scaffold, where ASCs aggregated to form multicellular spheroids. The two layers were securely combined and permeated into each other through a continuous and “soft” interface. In one group of animals, these bivalent scaffolds containing BMP-2 and preinduced ASC spheroids were implanted in OC defects created on the patellofemoral groove of the knee. Acellular scaffolds supplemented with BMP-2 or bare scaffolds were implanted in a second and third group of knee defects. At 12 weeks from implantation, both the cartilage and subchondral bone tissues were regenerated in the animals of the first group, whereas only subchondral bone was significantly regenerated in the second group, and poor tissue regeneration was found in the third group.

In a recent investigation, the implantation of 3D-printed full-thickness scaffolds was tested in OC defects generated in the trochlear groove of minipigs [123]. In this study, bilayer scaffolds were also designed for inducing ASC osteogenesis and chondrogenesis in the opposite sides. In particular, TCP incorporated into a PCL base was used to induce ASC osteogenic differentiation at the bottom, whereas decellularized bovine cartilage ECM was placed in the superficial side to promote ASC chondrogenic differentiation. In some scaffolds, an electrospun disk was inserted between the two layers, both to mimic the tidemark existing in the natural OC unit and to prevent blood vessel infiltration and ossification of the superficial part of the scaffold, where cartilage regeneration should take place. Five conditions were comparatively tested: (a) open lesion defects as a negative control; (b) the insertion of acellular scaffolds without tidemarks; (c) the insertion of acellular scaffolds with tidemarks; (d) the insertion of scaffolds seeded with autologous ASCs; and (d) the insertion of autologous explants as a positive control. In vivo evaluations after 4 months from implantation showed that the autologous explant provided the best results when compared with the other groups except for the ASC-seeded scaffolds, whose outcomes more closely resembled the positive control. In animals with open lesions, the repair tissue was mostly disorganized, whereas the insertions of the acellular scaffolds mainly facilitated subchondral bone regeneration. The acellular scaffold without a tidemark more significantly filled the lesion, suggesting that the tidemark inhibited native cell migration and infiltration into the cartilage-devoted portion of the scaffold. In these cases, however, very limited cartilage regeneration was detected. Indeed, MSCs from others sources have also been tested. Human umbilical cord blood MSCs were seeded in bivalent scaffolds consisting of HA and gelatin-based microcryogels to induce bone and cartilage regeneration, respectively [9]. In in vivo engrafted osteochondral defects in the femoral trochlear groove of dogs, these scaffolds achieved satisfactory levels of defect filling with newly formed tissue, showing a biphasic cartilage and bone structure.

The main results of the ASC-based strategies are summarized in Table 3.

## 7. Conclusions

As described in the investigations reported in the present paper, stem cells are being increasingly studied to develop therapeutic applications aimed at the treatment of skeletal tissue disorders, similar to many pathologies affecting other tissues and organs [37,124]. The target is to improve the quality of life of patients affected by diseases for which current therapies still provide poor results, as occurs for many bone and cartilage disorders. Due to their mesodermal origin, adult multipotent MSCs are the natural choice and are widely investigated for tissue engineering in this field. In addition, they are more suitable for applications in humans, minimizing legal, religious, ethical and safety issues. Among MSCs, ASCs have been increasingly investigated for their advantages and encouraging results that have been repeatedly reported by researchers worldwide both for human and animal models. In some cases, freshly isolated ASCs were tested; in other studies, expanded and/or predifferentiated ASCs were implanted; and in more complex strategies, ASCs seeded in a variety of supporting scaffolds were applied. For safety and regulatory restrictions, minimally manipulated ASCs were tested for clinical applications [125]. In fact, when MSC in vitro expansion is carried out, it may affect their biological properties and regenerative potential [126]. Adverse events and side effects associated with MSC therapy have been recently reviewed [127]. Cell processing, such as isolation, culturing and storage may influence cell population profile and differentiation, change protein expression and possibly induce negative effects. Chromosomal abnormalities may result from long-term cell culturing, and overdosed antibiotics in culture media may increase the risk of mycoplasma contamination. Adverse event manifestation may also depend on the patient’s individual phenotype, and possible complications should be considered when planning clinical trials. In a recent study, the conclusions of 19 meta-analyses regarding the efficacy and safety of treating primary knee OA with stem cells were analyzed. Based on the results reported, it was concluded that these meta-analyses could hardly provide a scientifically definitive assessment of the efficacy of stem cell treatment in this field [128]. However, investigations performed in vitro and in animal models already show ASCs’ enormous therapeutic potential. Overall, although some variability exists among the results reported, it can be assumed that the best levels of bone and cartilage regeneration are achieved using predifferentiated ASCs and scaffolds. One just has to hope that in the near future a wider number of these strategies may be safely translated from bench to bedside to improve the quality of life of more patients suffering pain and significantly reduced mobility.

## Figures and Tables

**Table 1:** Examples of ASC use for bone repair.

Cells	Study Design	Outcomes	References
ASCs overexpressing basic FGF	Intramuscular injection in a mouse model of femur fracture	Improved ASC engrafting, mineralization activity, angiogenesis and osteogenic differentiation. Accelerated bone repair	[68]
ASCs predifferentiated toward osteogenic linage and endothelial precursor cells	Subcutaneous implantation of osteogenic cell sheet and endothelial progenitor cell complexes in nude mice	Dense new ectopic bone tissue formation	[70]
	Implantation of osteogenic cell sheet and endothelial progenitor cell complexes in rabbits with calvarial defects	Satisfactory bone tissue reconstruction	
ASCs from osteoporotic mice and ASCs from healthy mice	Osteogenic differentiation in vitro of ASCs	Lower proliferation rate and osteogenic differentiation ability by osteoporotic ASCs	[40]
ASCs from aged and young mouse	In vitro tests of proliferation rate and osteogenic differentiation	Higher proliferation rate and differentiation ability by young ASCs	[71]
	Injection in bone marrow of osteoporotic mice	Improved bone regeneration and increased bone mineral density by young ASCs	
ASCs and/or BMSCs	Injections in rat calvarial defects	Improved bone formation, especially for injections of both cells	[72,73]
Fibrin-encapsulated ASCs in printed PCL scaffolds	TNF-α and PDGF treatment in osteogenic and vasculogenic medium	Improved new vessel formation and matrix mineralization at low doses of TNF-α	[76]
	Subcutaneous implant in athymic nude rats	Improved angiogenesis and bone tissue maturation	
Scaffold-assisted implantation of native or predifferentiated ASCs	Subcutaneous implantation in mice	Enhanced ectopic bone formation	[42,74,77,78,83]
Titanium scaffolds coated with cell matrix hydrogel ASCs	Implantation in a rat model of full-thickness mandibular defects	Improved bone regeneration and new bone formation	[85]
ASCs in 3D-printed PCL/TCP scaffolds functionalized with bone ECM	Implantation in dogs with mandibular defects	More pronounced ossification	[86]
ASCs in PRP/collagen scaffolds	Injection in mandibular osteoradionecrosis model of athymic rats	Enhanced bone preservation and deposition along with increased osteoblasts and decreased osteoclasts	[87]
Engineered ASCs for BMP-2 and/or VEGF release seeded in acellular bone matrix	Implantation in ulnar bone defects of minipigs	Accelerated repair of bone defects	[88]
Engineered ASCs overexpressing osterix in gelatin/VEGF-coated PCL scaffolds	Implantation in rats with calvarial defect	Improved ASC osteogenesis and bone repair	[90]
ASCs in gelatin microribbon-based microporous hydrogel supplemented with BMP-2	Injection in a mouse model of cranial defect	Enhanced ASC survival and good filling of the bone defect	[91]
Indirect cocultures of ASCs and osteoblasts in collagen-based 3D scaffolds	Implantation in rats with calvarial defects	Good levels of new bone formation and coverage ratio	[92]
ASCs seeded in methacrylated gelatin hydrogels	Effects of photobiomodulation on scaffold implantation in rats with biparietal bone defects	Significantly improved reconstruction of bone defects	[93]
ECM hydrogel supplemented with ASC-derived exosomes	Injection in a rat model of intervertebral disc degeneration	ECM regeneration along with decreased ECM catabolism and reduced metalloprotease activity	[94]
Autologous ASCs	Autograft in oncology patients and in patients with nonunion bone fractures	Verified procedure safety and ASC clinical efficacy	[95]
Osteoinducted ASCs and demineralized bone matrix	Transplantation in patients with long bone nonunion or tumor resection	Improved osteogenesis, re-established bone function with no significant adverse side effects	[96]

Abbreviations: ASCs: adipose-derived mesenchymal stromal cells; BMP: bone morphogenetic protein; BMSCs: bone marrow stromal cells; ECM: extracellular matrix; FGF: fibroblast growth factor; PCL: polycaprolactone; PDGF: platelet-derived growth factor; PLA: polylactic acid; PLGA: polylactic-co-glycolic acid; PRP: platelet-rich plasma; RUNX2: runt-related transcription factor 2; TCP: tricalcium phosphate; TNF-α: tumor necrosis factor-α; VEGF: vascular endothelial growth factor.

**Table 2:** Examples of ASC use for cartilage repair.

Cells	Study Design	Outcomes	References
SVF cells and PRP	Injection into the knee of OA mouse model	Improved regeneration of injured articular cartilage and joint movement	[101]
ASCs	Injection into the knee of OA rabbit model	Significant improvements in the quality of cartilage	[102]
Engineered ASCs to overexpress IGF-1	In vitro experiments	Overexpression of chondrocyte anabolic markers	[103]
	Injection into the knee of OA mouse model	Increased ASC survival and reduced cartilage degeneration	
Chondrocyte-like differentiated ASCs	Injection into the knee of OA rat model	Enhanced hyaline-like neocartilage formation and fibrosis reduction	[49]
ASCs or SVF cells in collagen I/III scaffolds	Implantation in Dutch milk goats with cartilage defects of medial condyles and trochlear grooves of the knee	Extensive expression of collagen type II, hyaline-like cartilage. High levels of regenerated subchondral bone	[104]
Autologous ASCs combined with hyaluronic acid, PRP and calcium chloride	Percutaneous injection in the knee of OA patients and into the femoral head of patients with osteonecrosis	Improved cartilage regeneration in OA patients. Improved bone formation in patients with osteonecrosis	[106]
Autologous ASCs	Intra-articular injection in the knee of OA patients	Pain relief and improved joint function without serious adverse events	[107,108]
Chondrocyte-like differentiated ASCs in cartilage-based scaffolds	Implantation in the knee of rabbits with cartilage defects	Defect filling with chondrocyte-like tissue with smooth surface, showing collagen type II expression and positive Alcian blue staining	[100]
ASCs in cartilage-based scaffolds	Implantation in the knee of rabbits with cartilage defects	Improved chondrogenic differentiation. Good levels of hyaline cartilage regeneration	[109]
ASCs or ASC secretome in biodegradable porous sponge cartilage scaffolds	Implantation in rabbits with femoral trochlea cartilage damage	Enhanced cartilage regeneration better achieved by ASCs than secretome	[110]
ASCs in calcium alginate gel	Implantation in a full-thickness hyaline cartilage defect at the patellofemoral joint in rabbits	Complete cartilage repair	[111]
ASCs in amnion membrane-based biomimetic injectable hydrogel	Intra-articular injection in OA rat model	Reduced inflammation. Improved chondroprotective effects and cartilage regeneration	[112]

Abbreviations: ASCs: adipose-derived mesenchymal stromal cells; IGF-1: insulin-like growth factor 1; OA: osteoarthritis; PRP: platelet-rich plasma; SVF: stromal vascular fraction.

**Table 3:** Examples of ASC use for osteochondral defect repair.

Cells	Study Design	Outcomes	References
3D scaffold-free constructs of autologous ASCs	Implantation in the patellofemoral groove of minipig models of OC defects	Increased hyaline cartilage formation and improved subchondral bone regeneration	[117]
3D scaffold-free constructs of allogeneic ASCs	Implantation in the trochlear groove of the knee in rabbit models of OC defects	Improved articular cartilage and subchondral bone regeneration	[119]
3D scaffold-free concentric bicylindrical constructs of autologous ASCs	Implantation in minipigs with OC defects in the groove of the knee	Improved tissue repair	[120]
Chondrocyte-like ASCs in chitosan/gelatin hydrogel. Osteoblast-like ASCs in cancellous bone	In vitro assembly of 3D bilayered scaffolds for simultaneous bone and cartilage regeneration	Enhanced ASC proliferation compared with cells seeded on either single scaffold	[121]
Chondrocyte-like ASCs and osteocyte-like ASCs in poly(l-glutamic acid)-based 3D bilayer scaffolds	Implantation in rabbit model with OC defects in the patellofemoral groove of the knee	Improved simultaneous cartilage and subchondral bone tissue regeneration	[122]
3D bilayer scaffolds with an electrospun disk separating superficial chondrocyte-like ASCs in decellularized bovine cartilage ECM and underlying osteoblast-like ASCs in PCL/TCP	Implantation in OC defects in the trochlear groove of minipigs	Good levels of defect filling mainly by subchondral bone. Limited cartilage repair in the superficial part	[123]
3D bilayer scaffolds consisting of HA and gelatin-based microcryogels, seeded with umbilical cord blood MSCs	Implantation in OC defects in the femoral trochlear groove of dogs	Satisfactory levels of defect filling with newly formed cartilage and bone tissue	[9]

Abbreviations: ASCs: adipose-derived mesenchymal stromal cells; ECM: extracellular matrix; HA: hydroxyapatite; MSCs: mesenchymal stromal cells; OC: osteochondral; PCL: polycaprolactone; TCP: tricalcium phosphate.

## Data Availability

Not applicable.

## Abbreviations

ALP	alkaline phosphatase
ASCs	adipose-derived MSCs
BMP	bone morphogenetic protein
BMSCs	bone marrow stromal cells
ECM	extracellular matrix
ELISA	enzyme-linked immunosorbent assay
FGF	fibroblast growth factor
IGF-1	insulin-like growth factor 1
MSCs	mesenchymal stromal cells
OA	osteoarthritis
OC	osteochondral
PCL	polycaprolactone
PCR	polymerase chain reaction
PDGF	platelet-derived growth factor
PLGA	polylactic-co-glycolic acid
PLA	polylactic acid
PRP	platelet-rich plasma
RUNX2	runt-related transcription factor 2
SOX 9	sex-determining region Y box 9
SVF	stromal vascular fraction
TCP	tricalcium phosphate
TGF-β	transforming growth factor beta
TNF	tumor necrosis factor
VEGF	vascular endothelial growth factor
